# X-Rays

**Published:** 1916-10

**Authors:** 


### X-Rays.

**Simple and Rapid Method of Obtaining Skiagrams.** Rivier and Dupoux, Archives d'Electricite Med., Aug., use ferrotype plates instead of glass. Very hard tubes are used (convenient as available for both fluoroscopy and radiography, 9 or 10 B with maximum of 2 milliamperes in the tubes, corresponding to a primary current of 6 or 7 amperes at 70 volts. The spark is 22 c.m. The exposure varies from 6 seconds for the hand to 60 for the cranium. All developers with metol-hydroquinine may be used if strong in bromid. The negative generally comes up in 2 minutes and the fixation of the negative in hyposulphite solution transforms it immediately into a positive, in 10-20 seconds. Washing and drying occupy 2 or 3 minutes. The whole process requires less than 10 minutes and, at ambulance stations, the plate can be attached to the patient.

